# Retraction: Molecular Mechanisms of Nanosized Titanium Dioxide–Induced Pulmonary Injury in Mice

**DOI:** 10.1371/journal.pone.0297254

**Published:** 2024-01-11

**Authors:** The PLOS ONE Editors

Following the publication of this article [1], multiple concerns were raised regarding the results and text. Specifically,

- Values in Tables 1–3 described as standard error (SE) consistently represent 5% of the mean.
- There is text overlap with previously published articles which do not share authors with [1]:
  ⚬ Substantial overlap in the Discussion section with [2], without attribution.
  ⚬ Minor overlap in the Introduction with [3], and in the Discussion with [4–7], without attribution.
- There are multiple instances of text overlap between the Methods and Results sections of [1] and other works by the same author group that were published first or were under consideration at the same time, including [8, 9, retracted in 10, 11, retracted in 12]. Text reuse was not cited or acknowledged in [1] and similar submitted manuscripts were not declared during the peer review process as is required by PLOS policy.
- Tables 1–3 contain letters described as indicating significant differences between groups, however it is not clear which groups these refer to and how these significance values were calculated. The statistical analysis methods were not reported in sufficient detail in the article or in the comments received in post-publication discussions.

The corresponding author stated that an error occurred in the calculation of SE values. They provided updated tables presenting mean ± standard deviation, as well as raw numerical data underlying the results presented in these tables.

The author stated that one-way ANOVA was used to compare groups in the tables. However, they did not clarify if a post-hoc test was used to compare individual groups, and their comments did not clarify the meaning of the letters in the tables or provide sufficient reporting of statistical analysis methods to meet the journal’s requirements.

In addition to the above comments, the corresponding author stated that the unit for OHdG in Table 3 should be ng/g, not mg/g.

In light of the above issues, the article does not meet the journal’s reporting and policy requirements and PLOS remains concerned about the validity and reliability of the reported quantitative results. Therefore, the *PLOS ONE* Editors retract this article. We regret that the issues were not identified before the article was published.

TZ agreed with the retraction. BL, YZ, QS, XS, YC, XW, SG, DT, MZ, XZ, LS, LW, FH, and MT either did not respond directly or could not be reached.
